# Pretreatment of Mice with Oligonucleotide prop5 Protects Them from Influenza Virus Infections

**DOI:** 10.3390/v6020573

**Published:** 2014-02-06

**Authors:** Kang Li, Zhe Zhou, Yu ou Wang, Juan Liu, Hai bao Zhao, Jing Yang, Sheng qi Wang

**Affiliations:** 1 Beijing University of Technology, No.100, Pingleyuan, Chaoyang District, Beijing 100124, China; E-Mail: caaslk@163.com; 2 Beijing Institute of Radiation Medicine, No.27, Taiping Road, Haidian District, Beijing 100850, China; E-Mails: zhouzhe@bmi.ac.cn (Z.Z.); kings108@163.com (Y.O.W.); liu458868056@126.com (J.L.); xuehaibaozong@163.com (H.B.Z.); 3 Henan University of Traditional Chinese Medicine, No.1 Jinshui Road, Zhengzhou, Henan 450008, China

**Keywords:** influenza A virus, host factor, antisense oligonucleotide, prop5

## Abstract

Influenza A virus is a successful parasite and requires host factors to complete its life cycle. Prop5 is an antisense oligonucleotide, targeting programmed cell death protein 5 (PDCD5). In this study, we tested the antiviral activity of prop5 against mouse-adapted A/FM/1/47 strain of influenza A virus in a mouse model. Prop5 intranasally administered the mice at dosages of 10 and 20 mg/kg/d at 24 h and 30 min before infection, provided 80% and 100% survival rates and prolonged mean survival days in comparison with influenza virus-infected mice (both *p* < 0.01). Moreover, viral titres in mice pretreated with prop5, at dose of 10 and 20 mg/kg/d, had declined significantly on day two, four, and six post-infection compared with the yields in infected mice (*p* < 0.05 or *p* < 0.01); lung index in mice pretreated with prop5 (20 mg/kg/d) had been inhibited on day six post-infection (*p* < 0.05). Western blotting and immunohistochemistry showed that prop5 could down-regulate the PDCD5 protein expression levels in lung tissues of infected mice. These data indicate that antisense oligonucleotide prop5 is a promising drug for prophylaxis and control influenza virus infections and provides an insight into the host-pathogen interaction.

## 1. Introduction

Influenza A virus (IAV) is an RNA virus of the family *Orthomyxoviridae* and causes contagious respiratory disease with potentially fatal threats in both animals and humans. IAV remains a major public health issue, in particular, emergence of the novel swine-origin pandemic influenza A (H1N1) pdm09 in Mexico [1] and influenza A (H7N9) virus in China in early 2013 [2]. Vaccination is one of the effective tools of antiviral therapy of influenza, though it takes several months to produce an available vaccine against a new virus strain [3]. During the 2009 flu pandemic, approximately 99% of novel pandemic H1N1 virus isolates exhibit resistance to adamantanes (amantadine and rimantadine) [4]. Continuous surveillance of oseltamivir-resistant influenza viruses remained necessary in Japan during the 2007–2009 influenza seasons [5], and in the United States during 2007–2008 [6]. Zanamivir-resistant influenza viruses were isolated from Southeast Asia and Australasia between 2006 and early 2008 [7]. Pharmacological targeting host factors, required for influenza virus propagation, proved an alternative therapeutic strategy to minimize the likelihood of the emergence of viral resistance [8]. Basing on genome-wide RNA interference screening, two teams identified 295 [8] and 287 [9] human host cellular factors involved in IAV replication, respectively. They further confirmed that inhibition of vATPase, CAMK2B, CLK1, and Cdkn1b blocked influenza virus replication [8,9].

Programmed cell death protein 5 (PDCD5), also designated TFAR19 (TF-1 cell apoptosis related gene-19), could enhance apoptosis in different tumor cells (e.g., HeLa, TF-1, MCG-803, and MCF-7) [10]. In our laboratory, two-dimensional electrophoresis and Western blotting demonstrated that levels of PDCD5 expression are up-regulated in human lung adenocarcinoma epithelial cells (A549) after IAV infection [11]. Overexpression of human *PDCD5* in transected A549 cells enhanced replication of IAV in infected cells. On the other hand, inhibition of PDCD5 reduced the spread of virus in A549 cell cultures (data not shown). Prop5, a 20-mer antisense oligonucleotide (ASODN) targeting *PDCD5* mRNA, has been validated to down-regulate PDCD5 expression in A549 cells and inhibit propagation of influenza A/jingfang/1/86 (H1N1) virus.

In this study, we investigated the anti-influenza virus A/FM/1/47 (H1N1) activities of the prop5 *in vivo*. Our results suggested that prop5 could down-regulate PDCD5 expression, while exhibiting a prophylactic effect on the mouse-adapted variant of influenza virus infected mice model. 

## 2. Results and Discussion

### 2.1. Prop5 Protected Animals from Influenza Virus Infections

**Figure 1:**
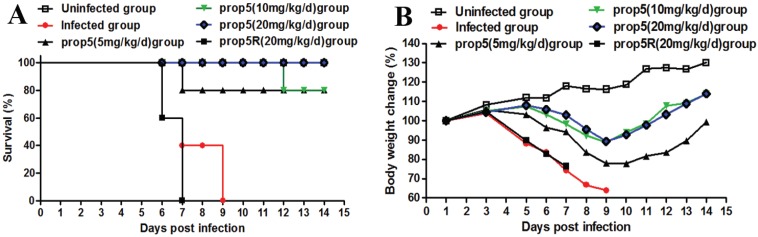
Effects of prop5 on survival rate and body weight loss in infected mice. BALB/c mice were pretreated with normal saline, prop5 (5, 10, 20 mg/kg/day, respectively) and prop5R (random sequence control of prop5, 20 mg/kg/day) dissolved in normal saline at 30 min and 24 h before infection. After drug pretreatment, each mouse was infected intranasally with 400 PFU of A/FM/1/47 (H1N1) in sterile normal saline or normal saline only (uninfected group) at a total volume of 20 μL. Mice (*n* = 10 in each group) were monitored for 14 days starting from virus infection. (**A**) Effects of prop5 on survival of infected mice; (**B**) Effects of prop5 on body weight loss of infected mice. Changes in body weight were based on the initial starting average weight at infection day. The results shown of body weight loss were the average values of body weights of living mice in each group.

To test the anti-influenza virus activity of prop5 *in vivo*, an A/FM/1/47 (H1N1) infected murine model was used. The results showed that prop5 (20 mg/kg/d) could completely protect mice from lethal challenge with the mouse-adapted variant of influenza virus. Eight out of ten mice survived when prop5 were given by intranasal administration at low doses of 5 and 10 mg/kg/d. On the contrary, prop5R (20 mg/kg/d) pretreated group and infected control mice all died. Meanwhile, prop5 at high dosage 20 mg/kg/d prevented lethality (Figure 1A). Mice pretreated with prop5 showed less clinical signs of disease and death, compared with infected mice controls and prop5R pretreated mice. Observation of average weight changes of each group manifested that prop5 could ease infected mice weight loss in a dose-dependent manner (Figure 1B).

**Table 1:** Effects of intranasal pretreatment with prop5 on A/FM/1/47 (H1N1) infected mice.

Pretreatment	No. of survives/total no.	MSD, (days)	Lung index (%)			Virus titre (Log_10_ TCID_50_/g lung ± SD)		
			Day 2	Day 4	Day 6	Day 2	Day 4	Day 6
Prop5 (5 mg/kg/d)	8/10	12.6 ± 3.13 *	0.79 ± 0.06	1.22 ± 0.64	1.15 ± 0.21	3.31 ± 0.09	3.03 ± 0.17 **	2.91 ± 0.52 **
Prop5 (10 mg/kg/d)	8/10	13.6± 0.89 **	0.83 ± 0.03	0.87 ± 0.17	0.98 ± 0.03	2.26 ± 0.02 *	2.81 ± 0.31 **	2.72 ± 0.44 **
Prop5 (20 mg/kg/d)	10/10	14.0 **	0.92 ± 0.07	0.95 ± 0.14	0.81 ± 0.07 *	1.41 ± 0.35 **	2.66 ± 0.27 **	2.40 ± 0.13 **
Prop5R (20 mg/kg/d)	0/10	6.6 ± 0.54	0.75 ± 0.05	1.17 ± 0.68	1.31 ± 0.43	3.48 ± 0.10	4.27 ± 1.19	4.92 ± 0.99
Infected control	0/10	7.4 ± 1.51	0.75 ± 0.08	1.38 ± 0.98	1.27 ± 0.24	4.12 ± 0.07	5.45 ± 0.50	5.74 ± 1.14
Uninfected control	10/10	14.0	0.67 ± 0.05	0.82 ± 0.06	0.80 ± 0.09	NA	NA	NA

Lung infection parameters were performed as described in Experimental Section. Significant differences from infected control: * *p* < 0.05, ** *p* < 0.01. MSDs, mean survival days; NA, not applicable; TCID_50_, 50% tissue culture infective dose.

### 2.2. Prop5 Decreased the Lung Infection Parameters

The effects on lung index and virus titres on two, four, and six day post-infection (d.p.i.) are shown in Table 1. Lung consolidation and weights increased in the infected mice as time was on 6 d.p.i.. Lung weights of mice in the group pretreated with prop5 at a dose of 20 mg/kg/d decreased significantly compared with the infected control at 6 d.p.i. (*p* < 0.05). Prop5 decreased virus production in lung tissues of pretreated mice in a dose-dependent manner. At 6 d.p.i., virus yields of the infected control groups were 4.92 log_10_TCID_50_/g of lung, which was higher than prop5 pretreated groups. With pretreatment of prop5 at doses of 5, 10, and 20 mg/kg/day, the mean virus yields were reduced to 2.91, 2.72, and 2.40 log_10_TCID_50_/g of lung (all *p* < 0.01), respectively.

### 2.3. Reduction of PDCD5 Protein Expression Levels by prop5 in BALB/c Mice

**Figure 2:**
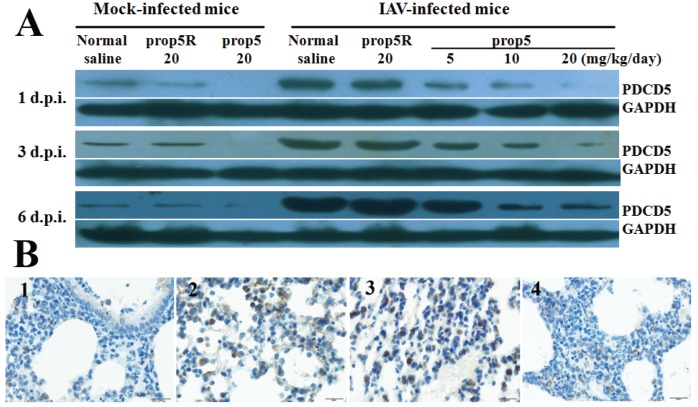
Prop5 down-regulated PDCD5 protein levels in mock-infected and influenza virus infected BALB/c mice (*n* = 5). Mice were pretreated with ASODNs and infected with A/FM/1/47 (H1N1), or received normal saline only (mock-infected mice) in the same manner as described above. (**A**) Expression of PDCD5 protein was inhibited by prop5 in mock-infected and influenza-infected mice lung tissue. At 1, 3, and 6 d.p.i., mice lung tissue samples were prepared for detection levels of PDCD5 by Western blotting. GAPDH served as the internal control; (**B**) Immunohistochemical staining was use to detected PDCD5 protein in mice lung tissue at 6 d.p.i. Weak expression of PDCD5 was detected in lung cytoplasm of mock-infected mice (1) and prop5 (20 mg/kg/d) pretreated mouse (4). Obvious expression and translocation into nuclei of PDCD5 were observed in lung of infected mouse (2) and prop5R (20 mg/kg/d) pretreated mouse (3). Bar = 20 μm.

We evaluated the PDCD5 protein expression levels in mock-infected and influenza virus infected mice pretreated with prop5. BALB/c mice were pretreated with ASODNs and infected with A/FM/1/47 (H1N1), or received normal saline only (mock-infected mice). We collected mock-infected and influenza-infected mice lung tissue samples at 1, 3, and 6 d.p.i. Western blotting analysis with specific PDCD5 antibody demonstrated prop5-inhibited expression of PDCD5 protein in mock-infected mice (Figure 2A, left). In influenza-infected mice, prop5 decreased overexpression of PDCD5 protein induced by IAV infection in a dose-dependent manner (Figure 2B, right). Immunohistochemistry was used to further assess inhibition of PDCD5 protein expression level by prop5 in influenza-infected mice at 6 d.p.i. As seen from Figure 2B, normal and prop5 (20 mg/kg/d) pretreated lung tissues showed weak expression level of PDCD5 in cytoplasm. PDCD5 in lungs of IAV infected mice and prop5R (20 mg/kg/d) pretreated infected mice obviously expressed and transposited into the nuclei. Taken together, these findings indicated that prop5 suppressed overexpression of PDCD5 induced by IAV infection. 

Apoptosis is regarded as an antiviral host response [12]. Paradoxically, IAV could induce apoptosis in numerous cell types both *in vivo* and *in vitro* [13,14,15,16]. This induced-apoptosis was to facilitate virus replication, and is closely associated with IAV virulence and pathogenicity [17,18,19]. Coevolving together with the host, IAV may acquire the ability to modulate anti- and pro-apoptotic signaling pathways to facilitate propagation, and, at the same time, to limit the cellular antiviral immune response [18,20,21]. PDCD5, a pro-apoptotic protein, took part in IAV vRNA synthesis [11]. Our data showed that prop5 efficiently suppressed overexpression of PDCD5 induced by IAV infection *in vivo*, and afforded protection against IAV in a murine model. Chen *et al.* proved that the PDCD5 played an important role in both caspase-3 activity and procaspase-3 cleavage [22]. IAV triggered caspase-3 activation with viral proteins, such as neuraminidase (NA) [23], hemagglutinin (HA) [24], nonstructural protein 1 (NS1) [25], and PB1-F2 [26], resulting in increased virus replication owing to enhanced migration of viral ribonucleoprotein complexes (RNPs) from the nucleus to the cytoplasm [17]. Studies showed inhibition of caspase-3 activation could suppress influenza virus propagation [17,27,28,29]. This might be one of the possible mechanisms of action of prop5 against IAV. The prophylactic treatment results of mice suggested that the antiviral activity of prop5 might be related with some other mechanism, such as an IFN-stimulating effect. However, because the random sequence (prop5R) control did not show the significant antiviral activity as prop5 *in vivo*, this IFN-stimulating effect should not be induced by the non-specific effect of prop5. Other mechanisms and possibilities of actions of prop5 against IAV need further studies.

## 3. Experimental Section

### 3.1. Virus, Cell, Mice, and ASODNs

A mouse-adapted variant of influenza virus A/FM/1/47 (H1N1) was grown in the allantoic cavity of 10-day-old specific pathogen-free embryonated chicken eggs (Merial-Vital Laboratory Animal Technology, Beijing, China) for 48 h at 35 °C. Virus production was determined by measuring haemagglutinin units. Madin-Darby canine kidney (MDCK) cells (ATCC, Manassas, VA, USA) were used for virus titration. Specific pathogen-free male BALB/c mice (18–20 g) were used to assay antiviral activity *in vivo*. Mice were bought from the Animal Centre of the Biomedical Institute of China (Beijing, China) and were housed in climatized colony rooms (22–26 °C, 60% humidity), with a 12 h light/dark cycle and free access to food and water. Mice were quarantined for 1 d prior to experiment. The experiments were reviewed and approved by the Animal Ethics Committee of the Beijing Institute of Radiation Medicine, in accordance with the regulations of the Beijing Administration Office of Laboratory Animals (No. SCXK-BJ-2009-0003). The DNA sequence of prop5 (5’-CCCTGTGCTTTGCTTCCTGT-3’) targeted 86–106 bp sequence of mRNA transcript of PDCD5 gene (Accession No.: NM_004708.2). Prop5R, random sequence, (5’-CTCTCTTTGTTCTTCGGCCG-3’), served as a control. Synthesis, modification, and purification of ASODNs were carried out as in a previous report [30].

### 3.2. Detecting Expression of PDCD5 Protein by Western Blotting and Immunohistochemical Staining

Equal quantities of proteins (20 μg/well) were separated by sodium dodecyl sulfate polyacrylamide gel electrophoresis and transferred onto polyvinylidene difluoride (PVDF) membranes (Millipore, Bedford, MA, USA). The PVDF membranes were incubated overnight at 4 °C with corresponding primary antibodies: anti-PDCD5 and anti-GAPDH (Proteintech, Chicago, IL, USA). After washing, membranes were incubated with peroxidase-conjugated secondary antibodies for 1 h at room temperature. Then, the membranes were incubated with enhanced chemiluminescent (ECL) substrate (Millipore) and exposed to autoradiography film in dark. 

Specimen slides (4 μm) were deparaffinized and rehydrated. Slides were immersed in sodium citrate buffer (pH 6.0) and boiled at 100 °C for 2 min. The slides were washed, blocked for endogenous peroxidase activity, and incubated with anti-PDCD5 antibody (Proteintech) at 4 °C overnight. After washing, sliders were incubated with HRP-conjugated anti-rabbit IgG for 30 min at room temperature. Sliders were incubated with 3,3-diaminobenzidine (DAB) substrate and counter-stained with hematoxylin. Negative controls were incubated with PBS during the primary antibody incubation step.

### 3.3. Antiviral Activity of prop5 *in Vivo*

Mice were anaesthetized with pentobarbital sodium and then infected with influenza virus A/FM/1/47 (H1N1) (400 PFU/mouse) by intranasal instillation. The mice received one of the following pretreatments: normal saline, prop5 (5, 10, 20 mg/kg/d, respectively), and prop5R (20 mg/kg/d). Saline solution containing prop5 or prop5R was given by intranasal administration at 30 min and 24 h before infection. 10 mice were used for each group of pretreatment and control. Parameters for determining pretreatment effects included prevention of death through 14 d, lessening of weight loss and improvement of mean survival days (MSDs).

### 3.4. Lung Infection Parameters

Mice were pretreated and infected in the same manner as described above. The lung parameters were assayed at 2, 4, and 6 d.p.i., with 4 mice from each group. The lung tissues were homogenized in medium at 1:10 (*w/v*), and centrifuged at 3,200 × g for 5 min to pellet debris. Serial 10-fold dilutions of the lung homogenate supernatant were added to MDCK cell monolayer, as described previously [31]. The monolayers in the wells were observed daily and scored for virus-induced CPE. Data of infectivity were expressed as the number of TCID_50_s per gram of lung tissue. In addition, the lung index was calculated as a parameter of inflammation or consolidation according to the following formula: lung index (%) = lung weight (g)/body weight (g) ×100%. The lung infection parameters were determined in parallel with these survival experiments.

### 3.5. Statistics Analysis

The data were expressed as means ± standard deviation (SD). MSDs, virus titers, virus copies number, and lung indexes were analyzed by the Student’s t-test (two tailed).

## 4. Conclusions

In conclusion, prop5 manifested anti-IAV activity at reducing death of infected mice, lessening weight loss, reducing viral load and titres, and preventing lung consolidation. Although further studies about the mechanisms of anti-virus activity, toxicology, and pharmacokinetic profiles are requested, this study provides a new insight into the host-pathogen interaction and an opportunity for the development of host-factor-directed antiviral therapies.
